# High-performance thin-layer chromatographic method for simultaneous determination of some Angiotensin II Receptor Antagonists with amlodipine in spiked human plasma with UV detection

**DOI:** 10.1186/s13065-026-01765-6

**Published:** 2026-03-23

**Authors:** Ahmed A. Khorshed, Fatma M. Abdelnaeem, Dalia M. Nagy, Mohamed Oraby, Sayed M. Derayea

**Affiliations:** 1 Department of Pharmaceutical Analytical Chemistry, Faculty of Pharmacy, Sohag University, Sohag, 82524 Egypt; 2 Analytical Chemistry Department, Faculty of Pharmacy, Minia University, Minia, 61519 Egypt

**Keywords:** Amlodipine, AIIRAs, HPTLC, Absorbance, Plasma

## Abstract

A simple, sensitive, precise, and cost-effective high-performance thin-layer chromatographic method has been developed and validated for the simultaneous determination of Amlodipine and some Angiotensin II Receptor Antagonist drugs, including Olmesartan, Telmisartan, Candesartan, Losartan, and Irbesartan, in spiked human plasma. The HPTLC analysis utilized a mobile phase consisting of toluene: ethyl acetate: methanol: acetone: acetic acid (6:1.5:1:0.5:1, v/v/v/v/v) on an aluminum-backed layer of silica gel 60 F254. Amlodipine demonstrated a linear relationship within the range of 60–600 ng/band, while the AIIRA drugs showed linearity within the range of 90–900 ng/band. The proposed method exhibited good linearity, with correlation coefficients (r) ranging from 0.9939 to 0.9998 for all five studied mixtures. The method was validated according to International Council for Harmonization guidelines for linearity, accuracy, precision, robustness, and detection and quantitation limits. Amlodipine and the investigated Angiotensin II Receptor Antagonist drugs were successfully detected and quantified in both bulk drug and plasma samples, yielding high recovery percentages and low standard deviation values. The greenness of the proposed HPTLC method has been evaluated using the standards of greenness profile (AGREE and GAPI) and Eco-Scale.

**Supplementary Information:**

The online version contains supplementary material available at 10.1186/s13065-026-01765-6.

## Introduction

Essential hypertension is characterized by an increase in blood pressure without an apparent cause, elevating the risk of cardiac, brain, and renal problems [1]. In developed nations, nearly 90% of the population faces a lifetime risk of developing hypertension (blood pressure greater than 140/90 mmHg). Hypertension is frequently associated with other cardiovascular risk factors, such as aging, obesity, insulin resistance, diabetes, and hyperlipidemia [2]. Drugs from various pharmacological classes with differing efficacy profiles have been developed to treat hypertension. These include calcium channel blockers (CCBs), angiotensin receptor blockers (ARBs), Angiotensin converting enzyme inhibitors, diuretics, and β-blockers [3]. One of the oldest classes of antihypertensive drugs is CCBs, which are a heterogeneous group of medications [3]. Amlodipine (AML), a lipophilic, long-acting, third-generation dihydropyridine calcium channel blocker (CCB), has been widely used for the past 20 years. It is renowned for its efficacy and safety, supported by strong evidence from large randomized controlled trials demonstrating its effectiveness in reducing cardiovascular events [4]. AML works by preventing calcium from entering cardiac and vascular smooth muscle cells, thereby lowering peripheral vascular resistance [5]. It is prescribed to treat angina and high blood pressure, with several randomized studies confirming its effectiveness in managing angina pectoris. Due to its long half-life, AML is typically dosed once daily, which is advantageous for patient compliance [3].

Angiotensin II Receptor Antagonists (AIIRAs) have proven to be highly effective antihypertensive medications with superior tolerability since their introduction into clinical practice in 1995. When combined with thiazide diuretics and dihydropyridine CCBs, AIIRAs exhibit synergistic effects in lowering blood pressure without increasing adverse event incidence. Moreover, they have demonstrated benefits on mortality and morbidity in chronic renal disease and heart failure, particularly in the presence of type 2 diabetes [6]. The studied AIIRAs in this paper include Olmesartan (OLM), Telmisartan (TLM), Candesartan (CAN), Losartan (LOS) and Irbesartan (IRB) are nonpeptide drugs that have been approved for use in the USA and Europe. A tetrazolo-biphenyl structure is shared by CAN, OLM, IRB and LOS, a benzimidazole group is shared by CAN and TLM [7].

**Fig. 1:**
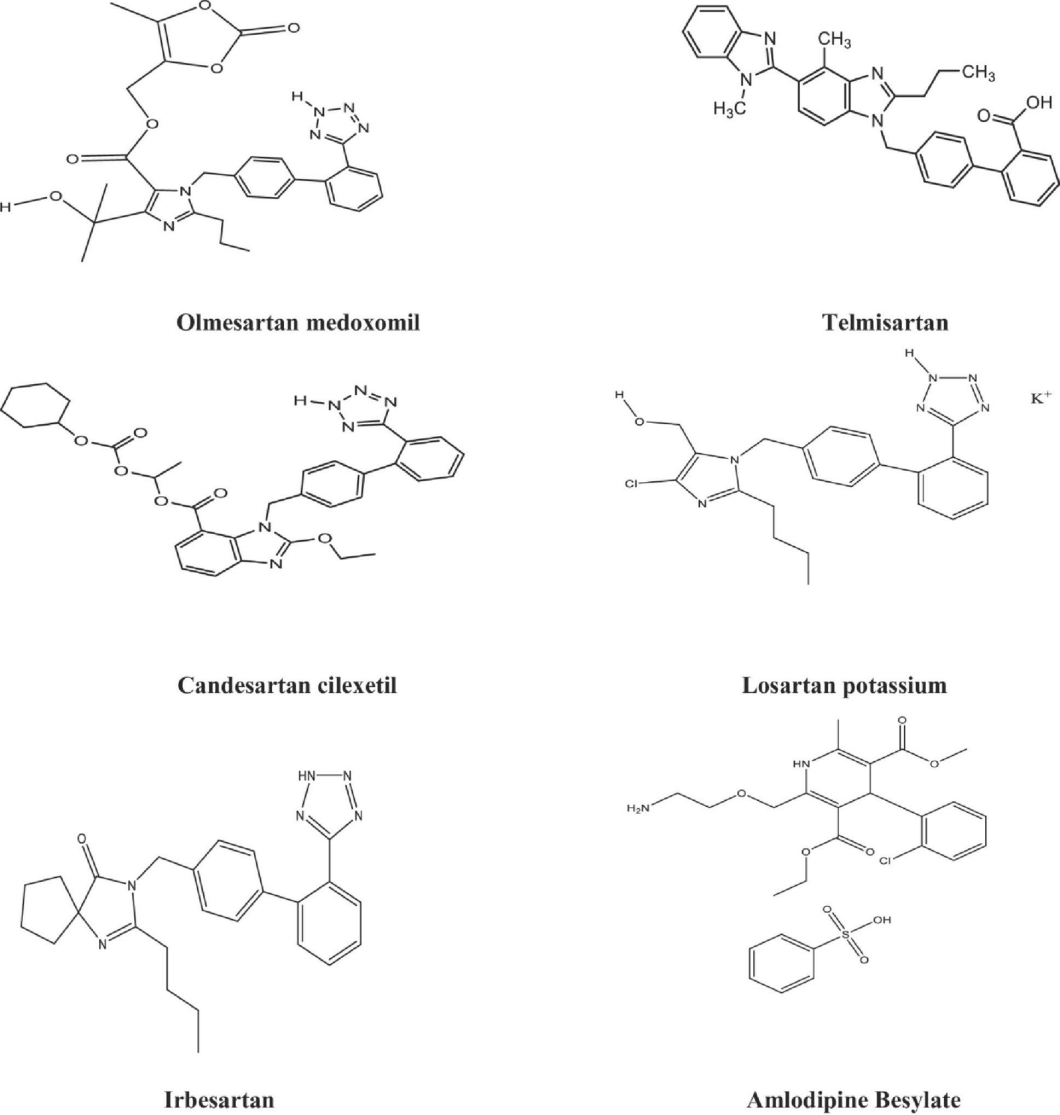
Chemical structures of the studied drugs

For most hypertensive patients, concurrent administration of two or more medications is essential to manage blood pressure and reduce risk factors [2]. AML is often co-administered with AIIRAs to enhance their efficacy in patients with grade 1–2 hypertension [8]. Combining AIIRAs such as OLM, TLM, CAN, LOS, and IRB with CCBs like AML effectively lowers and regulates blood pressure (Fig. 1) [8, 9]. AIIRAs have an antisympathetic effect, which significantly improves AML’s tolerance profile. They also reduce the likelihood of a heart rate increase with AML administration and partially mitigate the dose-limiting side effect of AML, which is peripheral edema [10].

AML and the studied AIIRAs have been analyzed using various methods, including spectrophotometry [11, 12], spectrofluorometry [13–15] and square wave voltammetry [16–18] but these techniques are typically applied to dosage forms rather than biological fluids. Several chromatographic techniques have been developed for the determination of the studied drugs, including HPLC [19, 20], GC-MS [21, 22] and HPTLC [23–25]. Most of the reported methods for their determination in biological fluids depended on using HPLC [20, 26] which uses large volume of expensive solvents. HPTLC is a widely used analytical technique due to its advantages, including low solvent usage, batch analysis, and rapid analysis. This technique allows for the concurrent measurement and identification of multiple compounds [27–30].

None of the previously published methods employ HPTLC with UV detection suitable for the simultaneous determination of various AIIRAs and AML. Thus, there is a need to establish and validate a simple, rapid, and economical HPTLC method for their simultaneous determination in spiked plasma using reflectance/absorbance mode. For the first time, a new High-Performance Thin Layer Chromatography (HPTLC) system with UV detection had to be developed and tested for their simultaneous determination. In contrast to earlier studies that restricted use to pharmaceutical formulations, the present method’s great sensitivity allowed for the identification of the compounds under study in plasma samples. The current process offers significant advantages in terms of saving time, solvents, effort, and cost, as five mixtures can be analyzed on the same plate with the same mobile phase. The proposed method was validated according to ICH standards [31].

## Experimental

### Apparatus

A Camag-TLC system (CAMAG, Muttenz, Switzerland) with Scanner III was used for the chromatographic analysis. WinCATS version 1.4.4.6337 software was used to control the TLC. The radiation source used in the reflectance/absorbance mode was a high-pressure deuterium and halogen tungsten lamp. The scanning speed was set at 20 mm/s, and the scanner had a slit measuring 3 × 0.45 mm. Application of the sample on the plate was done using a Linomat V auto-sampler and a Hamilton syringe (100 µl, Bonaduz, Switzerland) with a mild nitrogen stream. Under rising conditions, the plate was developed in a twin-trough chamber with dimensions of 27.0 × 26.5 × 7.0 cm.

### Chemicals and materials

Global Napi Pharmaceutical Co. kindly provided Amlodipine besylate, Candesartan cilexetil, and Losartan potassium (6th of October, Egypt). Sigma Pharmaceutical Company in Qewaisna, Egypt provided Telmisartan. Chemipharm Pharmaceutical Co. kindly supplied Olmesartan medoxomil (6th of October, Egypt). Irbesartan was graciously provided by Memphis Pharmaceutical Co. the (Cairo, Egypt). HPTLC measures were used to examine the pharmaceuticals under investigation for their purity. The purity values of the studied drugs were 98.21 ± 1.22, 98.55 ± 1.54, 98.09 ± 0.89, 98.32 ± 1.45, 98.56 ± 0.75 and 98.66 ± 1.48 for losartan, irbesartan, Telmisartan, Olmesartan and amlodipine respectively. The following solvents of analytical grade (toluene, ethyl acetate, methanol, acetone, and acetic acid) were obtained from El-Nasr Pharmaceutical Chemicals (Abo-Zaabal, Cairo, Egypt). Tablets used in this paper were obtained from Abdin pharmacy, Sohag, Egypt. Erastapex^®^, contain 40 mg Olmesartan and 10 mg Amlodipine, Chartoreg CO^®^, contain 40 mg Telmisartan and 10 mg Amlodipine, Candalkan^®^, contain 16 mg Candesartan, Amosar^®^, contain 100 mg Losartan, Aprovel^®^, contain 300 mg irbesartan and Norvasc^®^, contain 10 mg Amlodipine.

### Standard solution preparation

Stock solutions containing 1.0 mg/ml of the investigated medications (OLM, TLM, CAN, LOS, IRB, and AML) were made by putting 10 mg of each drug into 10 ml test tubes, sonicating the mixture for 10 min, and then adding more methanol to make the final volume of 10 ml. The examined AIIRAs were combined with AML. Next, working standard solutions (30, 50, 100, 140, 200, and 300 µg/ml) of the investigated AIIRAs medicines and (20, 40, 80, 120, 160, and 200 µg/ml) of AML were prepared by diluting stock solutions of each medication with methanol. Using a syringe, a 3 µl aliquot of the working solutions that had been created was applied to the silica gel plate, yielding final concentrations of (90, 150, 300, 420, 600, 900 ng/band) of the studied AIIRAs drugs and (60, 120, 240, 360, 480, 600 ng/band) of AML.

### Preparation of pharmaceutical tablet dosage form solutions

Ten tablets were accurately weighted and finely powdered. An amount of powder equivalent to 10 mg of each drug was transferred into a 10-ml volumetric flask, about 7 ml methanol was added, sonicated for 15 min, completed with the same solvent to the mark, shaken well for 10 min, filtered and first portion of the filtrate was rejected. Suitable volumes of the studied drugs were diluted with methanol to obtain suitable concentrations that lie in the linear range of each drug and completed as described in the chromatographic conditions.

### Plasma samples preparation

Plasma isolation from blood was conducted at Misr Hospital (Sohag, Egypt). Blood samples (5 mL per subject) were withdrawn from 10 healthy adult volunteers (age range: 20–40 years, who had not taken AIIRAs or other medications) through the forearm vein and immediately transferred into heparinized tubes. A total of 0.5 mL plasma per subject was used for method development and validation (spiking and analysis). The volunteers provided written consent for their samples to be utilized and were informed about the experiment’s objectives. The plasma collection process adhered to the guidelines set forth by the Declaration of Helsinki.

A plasma sample of 150 µl was spiked with an equal volume (150 µl) of a specific concentration of the medications under study (AIIRAs) and 150 µl of a specific concentration of AML standard solutions in an Eppendorf, Hamburg, Germany centrifuge tube. The mixture was then vortexed for 2 min. Addition of 300 µl of acetonitrile, followed by a 5-min vortex mix, and a 15-min centrifugation at 14,000 rpm at − 4 °C. After extracting the clear supernatant from each centrifuge tube, the suggested methodology listed under the “Chromatographic conditions” was used to the analysis. Three distinct concentrations covering the linear range of each medication were used in six replicates for this technique.

### Chromatographic conditions for the separation of the studied mixtures

The separation was performed on silica gel 60 F254 HPTLC plates (Merck, Darmstadt, Germany) with a stationary phase thickness of 200 μm and a particle size of 5 μm. The plates’ exceptional purity and clarity were ensured by washing them with methanol prior to development. Plates were cut into 20 × 6 cm pieces with a solvent front at 5 cm. A mobile phase comprising 10 milliliters of Toluene: ethyl acetate: methanol: acetone: acetic acid (6: 1.5: 1: 0.5: 1, v/v/v/v/v) was saturated for 20 min at room temperature in order to separate the combinations under study. After development and drying, the plates were scanned at a specific wavelength for each mixture in a single run to detect both drugs. The detection wavelengths were set at 244 nm for the AML–OLM Fig.S1 and AML–CAN mixtures, 247 nm for the AML–TLM Fig.S2 mixture, and 254 nm for the AML–LOS Fig.S3 and AML–IRB mixtures Fig.S4.

## Results and discussion

Hypertension is multifactorial disease and in recent years, the use of antihypertensive drug combinations with complementary mechanisms has increased significantly [32].

As all the previously documented HPTLC methods with UV detection were established for only one mixture and restricted to application in pharmaceuticals rather than plasma samples due to low sensitivity [33–35]. There is a pressing need to create a new HPTLC method with UV detection for the determination of the studied mixtures in spiked human plasma with acceptable levels of sensitivity. This current work provides a simple, quick, and sensitive method for simultaneous determination of AML and the studied AIIRAs “using UV detection” in spiked human plasma.

**Fig. 2:**
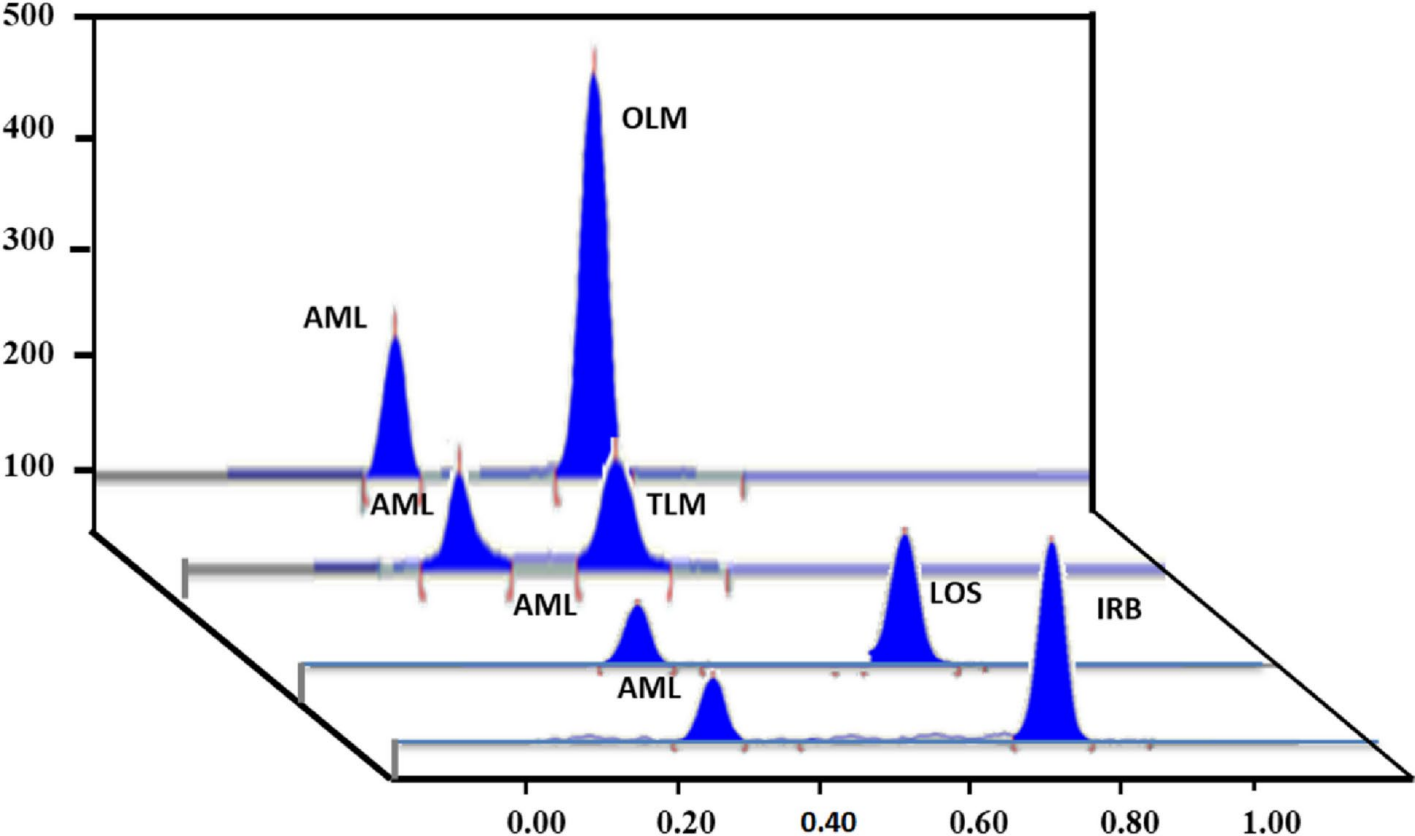
HPTLC densitogram of mixture containing. 600 ng/band of AML and 900 ng/band of OLM. 480 ng/band of AML and 600 ng/band of TLM. 270 ng/band of AML and 270 ng/band of LOS. 420 ng/band of AML and 420 ng/band of IRB

The use of mobile phase of Toluene: ethyl acetate: methanol: acetone: acetic acid (6: 1.5: 1: 0.5: 1, v/v/v/v/v), gave well-separated, compacted and highly resolved bands for all the studied drugs, (0.50, 0.40, 0.62, 0.69, 0.73 ± 0.001 and 0.22 ± 0.002) for OLM, TLM, CAN, LOS, IRB and AML, respectively Fig. 2. Each mixture was detected in single run using one scanning wavelength as mentioned before. This is beneficial as it saves time, money, effort and solvents.

### Method development

**Fig. 3:**
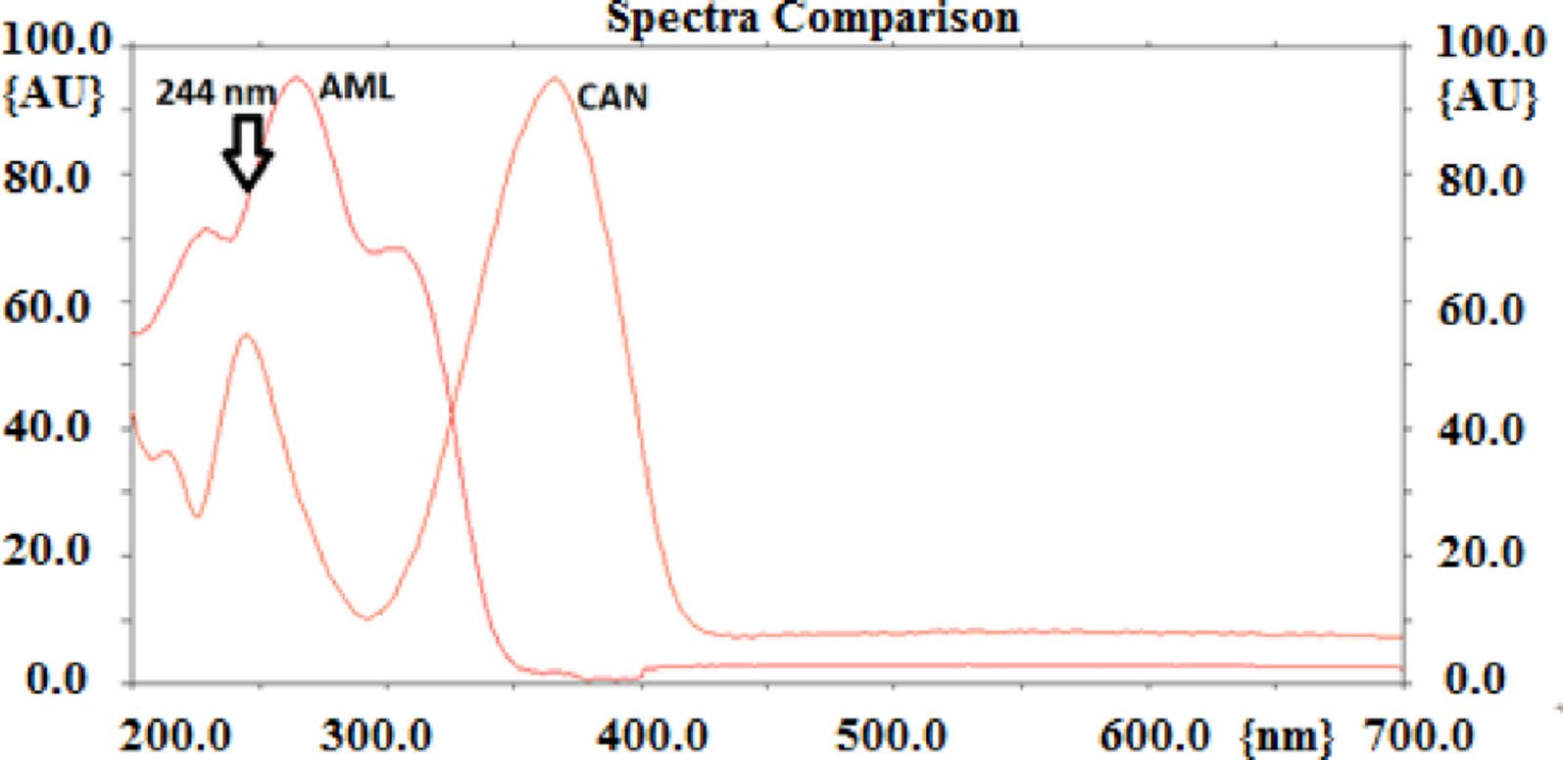
Absorption spectra of amlodipine and candesartan

The studied AIIRA’s exhibit a greater antihypertensive effect when taken in combination with AML [8, 9]. Unfortunately, the spectra of the studied drugs are overlapped with AML as shown in Fig. 3 for AML with CAN as a representative example. Numerous methods for the simultaneous determination of AML with the studied AIIRA drugs using derivative spectrophotometry or ratio derivatives have been reported [11, 12, 36]. However, these methods have significant drawbacks, such as low signal, poor sensitivity, an inconvenient signal-to-noise ratio, poor robustness, and susceptibility to potential interferences from excipients [37–39]. HPTLC technique allows simultaneous determination of the mixed drugs at one maximum wavelength without any interference from each other or from the combined excipients as they are separated at different R_f_ values [40–43]. The current study used single detection wavelength for each mixture of the studied five mixtures utilizing the same chromatographic system (stationary and mobile phases) for the separation and simultaneous detection and quantification of the studied AIIRAs and AML.

Several trials were carried out to select the most suitable mobile phase for simultaneous determination of AML with each of the studied AIIRAs drugs, these are summarized in Table S1.

**Fig. 4:**
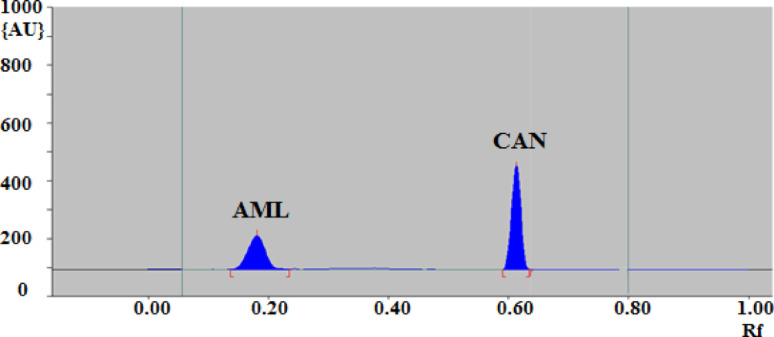
HPTLC densitogram of mixture containing 600 ng/band of AML and 900 ng/band of CAN

Fortunately, mixture of Toluene: ethyl acetate: methanol: acetone: acetic acid (6: 1.5: 1: 0.5: 1, v/v/v/v/v) were used. The selected mobile phase should be able to give compact spots of the drugs with suitable R_f_ values. The obtained Rf values for the studied drugs are (0.50, 0.40, 0.62, 0.69, 0.73 ± 0.001 and 0.22 ± 0.002) for OLM, TLM, CAN, LOS, IRB and AML respectively. Figure 4 shows HPTLC densitogram of AML and CAN using Toluene: ethyl acetate: methanol: acetone: acetic acid (6: 1.5: 1: 0.5: 1, v/v/v/v/v) as a mobile phase.

### Validation of the method

The developed method was fully validated according to the ICH guidelines [31] and complied for linearity, accuracy, and precision, detection and quantification limits, and robustness. The statistical analysis was carried out with the 95% confidence level.

#### Linearity

**Fig. 5:**
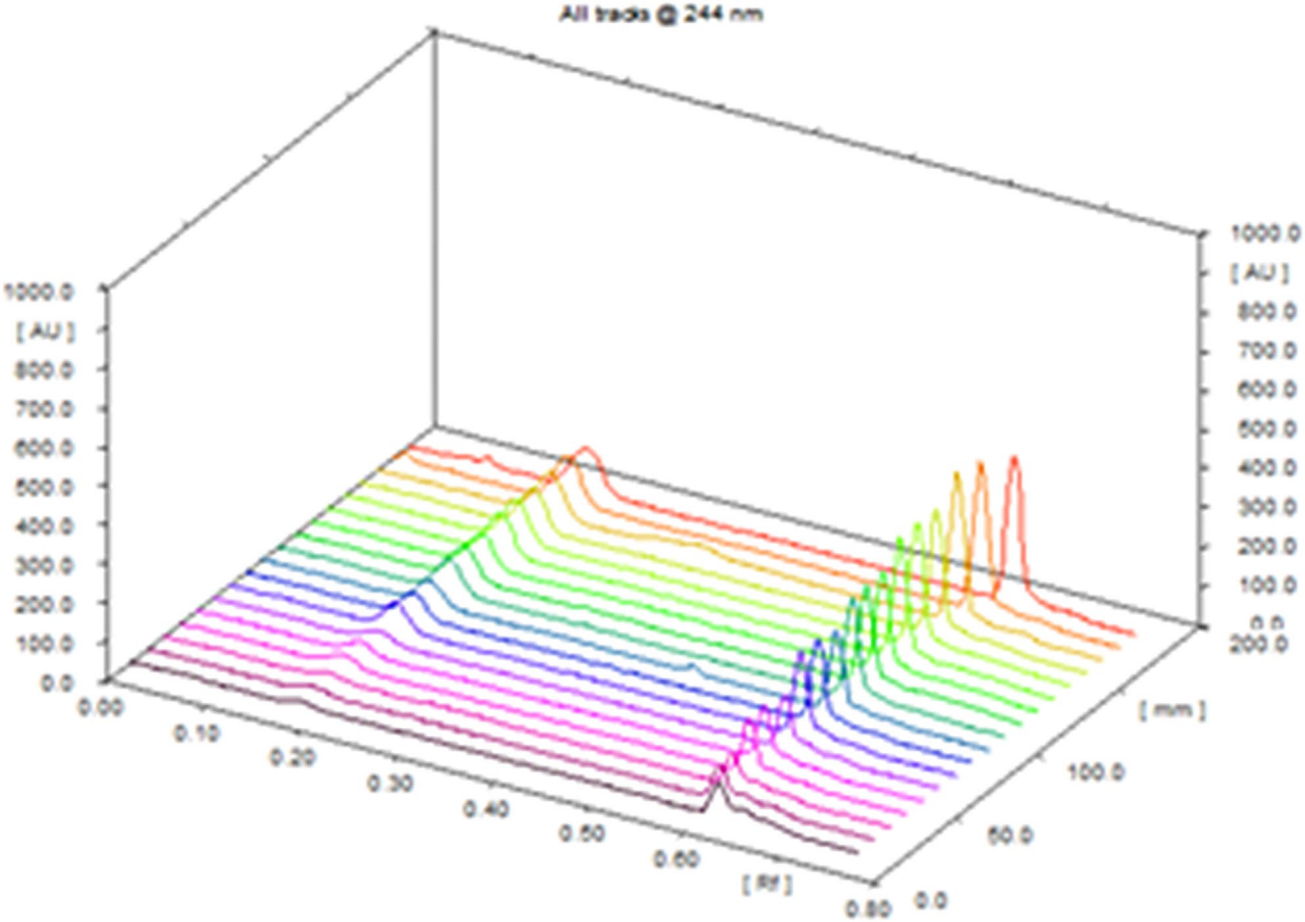
Three-dimensional graph showing recorded intensities against R_f_ values for (60–600 ng/band) for AML, and (90–900 ng/band) for CAN measured by reflectance/absorbance mode at 244 nm

Six concentration levels (three replicates for each) for all of the studied drugs were selected to construct the calibration curves. Calibration parameters are given in (Table 1) showing high correlation from the calculated correlation coefficients (r) between the investigated concentrations and the corresponding obtained peak areas. They were in the range 0.9939–0.9998, expressing good linear relationship of the suggested method. Figure 5 shows three dimensional densitogram for mixture of AML and CAN as a representative example over the ranges of (60–600 ng/ band) and (90–900 ng/band) respectively.

**Table 1:** Statistical data for the simultaneous HPTLC determination of some AIIRAs–Amlodipine mixtures with UV detection

Parameters	OLM–AML		TLM–AML		CAN–AML		LOS–AML		IRB–AML	
	OLM	AML	TLM	AML	CAN	AML	LOS	AML	IRB	AML
Linear range (ng/band)	90–900	60–600	90–900	60–600	90–900	60–600	90–420	60–480	90–480	60–480
Correlation coefficient, r	0.9984	0.9939	0.9998	0.9986	0.9992	0.9992	0.9968	0.9971	0.9993	0.9984
Determination Coefficient, r2	0.9969	0.9880	0.9996	0.9972	0.9984	0.9985	0.9937	0.9942	0.9986	0.9968
Slope ± SD (a)	6.66 ± 0.18	5.81 ± 0.32	5.44 ± 0.05	5.75 ± 0.15	2.92 ± 0.06	3.32 ± 0.06	4.23 ± 0.15	2.27 ± 0.07	6.86 ± 0.11	2.15 ± 0.05
Intercept ± SD (a)	414.35 ± 48.93	-74.61 ± 25.28	7.02 ± 26.90	− 249.10 ± 21.77	1224.48 ± 20.42	87.96 ± 13.37	397.03 ± 27.71	134.85 ± 9.70	80.82 ± 34.13	43.56 ± 10.40
RF	0.50 ± 0.001	0.20 ± 0.002	0.41 ± 0.001	0.20 ± 0.002	0.61 ± 0.001	0.20 ± 0.002	0.62 ± 0.001	0.20 ± 0.002	0.76 ± 0.001	0.20 ± 0.002
LOD (ng/band)	22.04	13.05	14.83	11.36	20.98	12.08	19.65	12.82	14.93	14.51
LOQ (ng/band)	73.46	43.51	49.44	37.86	70.05	40.27	65.51	42.73	49.75	48.37

(a) Average of three determinations

#### Limits of detection and quantification

The calculated LOD and LOQ for the studied drugs using the proposed method indicated high sensitivity (Table 1). They are calculated from the standard deviation of the intercept’s response and the slope of the calibration curve using these equations: LOD = 3 Ϭ/ b and LOQ = 10 Ϭ/ b, where b denotes the slope of the calibration curve and Ϭ denotes the standard deviation of the intercept.

The calculated LOD and LOQ values of the proposed method with UV detection were found to be, respectively, (13.05, 22.04 ng/band) and (43.51, 73.46 ng/band) for the AML–OLM mixture, (11.36, 14.83 ng/band) and (37.86, 49.44 ng/band) for the AML–TLM mixture, (12.08, 20.98 ng/band) and (40.27, 70.05 ng/band) for the AML–CAN mixture, (12.82, 19.65 ng/band) and (42.73, 65.51 ng/band) for the AML–LOS mixture, and (14.51, 14.93 ng/band) and (48.37, 49.75 ng/band) for the AML–IRB mixture.

#### Accuracy

The results obtained in (Table 2) by carrying out six times measurements of three different concentrations of each drug were satisfactory as indicated, the obtained recoveries ranged from 97.92 to 103.76 which indicated good accuracy of the suggested method. As the proposed method’s great accuracy was judged from the closeness of the recovery percentages to 100% with low values of standard deviation.

**Table 2:** Evaluation of the accuracy of the proposed HPTLC method with UV detection for simultaneous determination of the studied AIIRAs–Amlodipine mixtures

Studied mixtures	AIIRAs OLM, TLM, CAN, LOS, IRB			AML		
	Conc. (ng/band)	Amount found (ng/band)	% Recovery (a) ± SD	Conc. (ng/band)	Amount found (ng/band)	% Recovery (a) ± SD
OLM–AML	150	146.88	97.92 ± 1.06	120	120.44	100.36 ± 2.33
	300	302.23	100.74 ± 1.91	240	236.12	98.38 **±** 1.56
	600	602.56	100.43 ± 0.99	480	472.65	98.47 **±** 1.09
TLM–AML	150	152.20	101.47 ± 2.16	120	118.97	99.15 ± 1.14
	420	417.09	99.31 ± 0.89	360	354.80	98.56 ± 2.08
	900	896.69	99.63 ± 1.05	600	608.89	101.48 ± 0.99
CAN–AML	150	147.01	98.00 ± 2.57	120	118.76	98.96 ± 1.57
	420	430.37	102.47 ± 1.82	360	358.96	99.71 ± 2.01
	900	904.12	100.46 ± 2.02	600	600.67	100.11 ± 2.47
LOS–AML	120	119.85	99.87 ± 2.60	90	92.01	102.24 ± 2.54
	270	276.11	102.26 ± 0.66	270	272.39	100.89 ± 2.19
	420	411.81	98.05 ± 1.13	480	486.65	101.38 ± 1.51
IRB–AML	120	124.52	103.76 ± 2.44	90	91.37	101.52 ± 2.83
	270	275.97	102.21 ± 2.58	270	274.16	101.54 ± 2.03
	420	424.22	101.01 ± 1.77	420	416.02	99.05 ± 1.17

(a) Average of six determinations at each concentration level

#### Precision

**Fig. 6:**
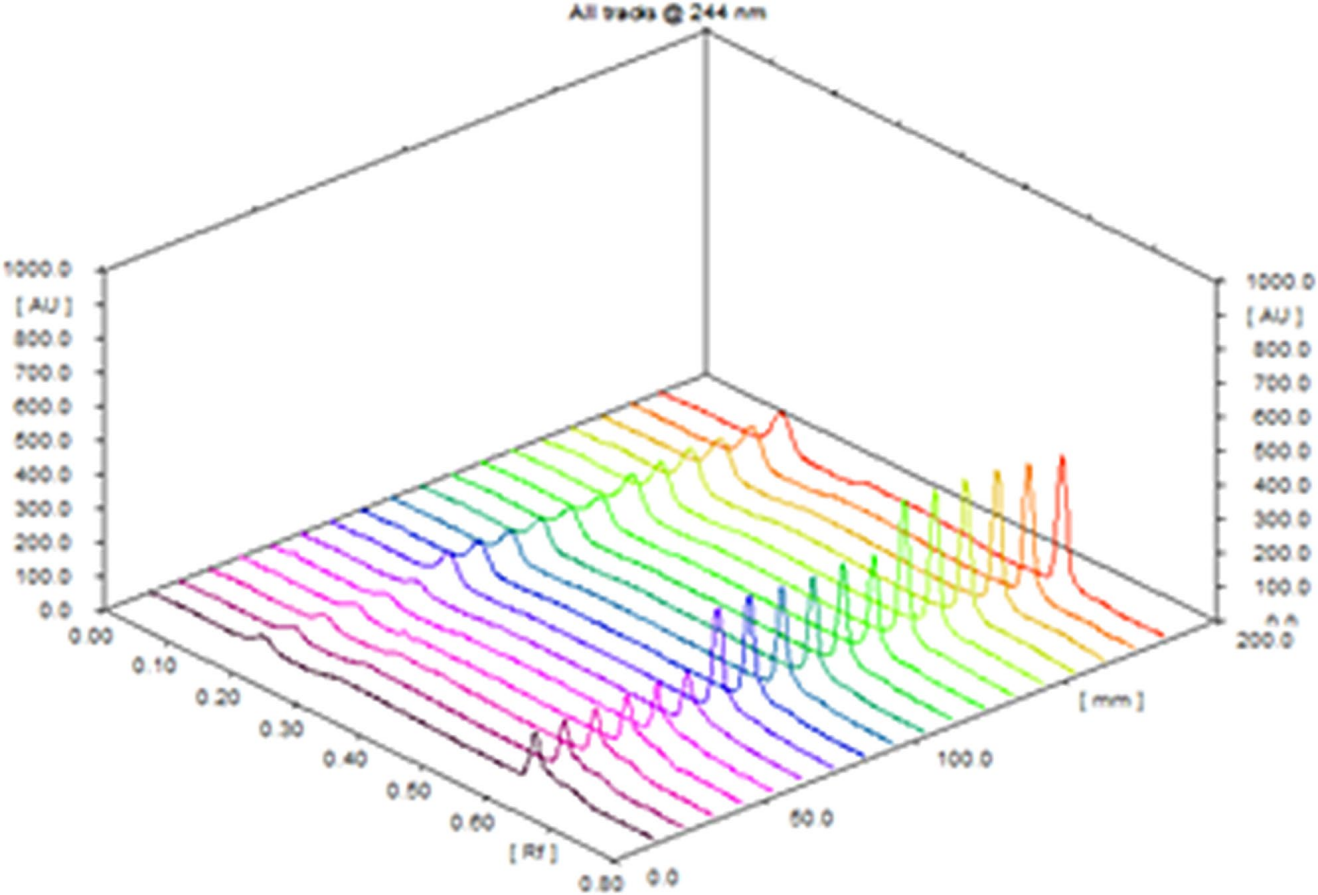
Three-dimensional graph showing precision for three concentrations of (120–360–600 ng/band) for AML, and (150–420–900 ng/band) for CAN measured by reflectance/absorbance mode at 244 nm

Intra and inter-day precision were expressed as percent relative standard deviation (% RSD) the results were given in (Table 3). For all concentration levels, the RSD didn’t exceed 2.85% indicating good precision of the proposed method (Fig. 6) they assessed by analyzing six replicates’ measurements of three different concentrations of each drug intra-daily and in three distinct days.

**Table 3:** Precision of the proposed HPTLC method with UV detection for simultaneous determination of the studied AIIRAs– Amlodipine mixtures at the intra- and inter-day levels

Studied mixtures	AIIRAs OLM, TLM, CAN, LOS, IRB			AML		
	Conc. (ng\band)	Intra-day RSD %(a)	Inter-day RSD %(b)	Conc. (ng\band)	Intra-day RSD %(a)	Inter-day RSD %(b)
OLM–AML	150	2.29	1.69	120	0.99	2.35
	300	0.69	1.30	240	1.71	1.26
	600	1.13	0.96	480	1.17	1.39
TLM–AML	150	2.02	2.34	120	2.55	2.21
	420	0.90	1.55	360	2.77	2.10
	900	1.04	1.27	600	2.64	1.30
CAN–AML	150	2.40	2.23	120	1.46	2.39
	420	1.18	2.26	360	2.26	1.89
	900	2.06	2.23	600	2.14	1.70
LOS–AML	120	0.98	1.12	90	2.09	2.25
	270	2.07	2.39	270	1.61	1.99
	420	1.11	1.09	480	1.59	1.88
IRB–AML	120	1.88	2.34	90	2.80	2.83
	270	1.27	2.85	270	2.22	2.00
	420	1.55	1.28	420	1.37	2.46

(a) Average of 6 determinations at each concentration level

(b) Average of 18 determinations at each concentration level over three distinct days

#### Robustness

A small variation in the proposed method parameter such as composition of the mobile phase, scanning wavelength, and saturation time, it was found that there was no significant effect on the method performance as given in (Table 4) so the method is considered to be robust.

**Table 4:** Results for the investigation of the robustness of the proposed HPTLC method with UV detection for simultaneous determination of the studied AIIRAs–amlodipine mixtures

Parameters	OLM–AML		TLM–AML		CAN–AML		LOS–AML		IRB–AML	
	OLM	AML	TLM	AML	CAN	AML	LOS	AML	IRB	AML
*Optimum parameters*	100.43 ± 0.99	100.36 ± 2.33	101.47 ± 2.16	99.15 ± 1.14	98.00 ± 2.57	98.96 ± 1.57	102.26 ± 0.66	100.89 ± 2.19	101.01 ± 1.77	99.05 ± 1.17
*Composition of the mobile phase* Toluene: ethyl acetate: methanol: acetone: acetic acid (5.8: 1.7: 1: 0.5: 1, v/v/v/v/v)	98.13 ± 1.15	99.26 ± 2.56	101.52 ± 1.22	100.67 ± 1.37	97.82 ± 2.28	98.44 ± 1.08	99.10 ± 0.89	101.35 ± 2.30	102.07 ± 1.49	100.73 ± 1.86
Toluene: ethyl acetate: methanol: acetone: acetic acid (5.8: 1.5: 1.1: 0.5: 1.1, v/v/v/v/v)	98.71 ± 2.13	100.53 ± 1.25	101.54 ± 1.78	99.43 ± 0.69	98.60 ± 1.70	101.71 ± 1.46	102.36 ± 1.38	99.02 ± 2.47	97.30 ± 2.36	101.41 ± 1.57
Toluene: ethyl acetate: methanol: acetone: acetic acid (6: 1.5: 0.8: 0.7: 1, v/v/v/v/v)	102.33 ± 1.70	101.47 ± 1.52	98.70 ± 2.53	101.45 ± 1.36	99.17 ± 0.79	98.53 ± 1.22	100.78 ± 1.59	102.71 ± 2.46	99.60 ± 1.58	98.15 ± 1.47
Scanning wavelength,										
242	101.44 ± 1.56	101.18 ± 1.44			99.47 ± 1.82	100.39 ± 1.68				
246	98.25 **±** 2.17	99.38 **±** 1.30			101.26 ± 1.57	98.36 ± 2.26				
245			97.56 ± 1.66	99.46 **±** 1.36						
249			99.34 **±** 2.10	100.57 **±** 1.48						
252							102.61 ± 1.50	98.36 ± 1.77	100.91 ± 2.34	101.26 ± 1.89
256							101.83 ± 1.56	97.91 ± 0.78	102.15 ± 2.41	100.77 ± 1.76
Saturation time										
18 min	98.56 ± 1.44	101.61 ± 1.40	100.72 ± 2.23	102.17 ± 0.86	101.66 ± 1.57	100.83 ± 1.19	97.60 ± 1.99	100.49 ± 2.16	101.67 ± 1.91	98.77 ± 1.43
22 min	100.48 ± 2.18	102.51 ± 1.97	99.37 ± 1.74	99.50 ± 1.59	98.46 ± 1.63	101.49 ± 2.16	100.85 ± 1.71	101.53 ± 1.48	101.49 ± 1.57	97.43 ± 1.75

(a) Average of six determinations

### Application of the proposed HPTLC method in pharmaceutical dosage form

The proposed HPTLC method with UV detection was applied for the determination of all the studied mixtures in pharmaceutical dosage form. The results obtained by average of six determinations of both the proposed and reported methods. The results obtained in Table 5 were validated by comparison with the results that were obtained using the previously reported methods for the determination of AML and the studied AIIRAs drugs. It was observed that there was no significant difference between the results obtained by the proposed methods and the reported methods as indicated by t- and F-tests.

**Table 5:** Application the proposed HPTLC method with UV detection for simultaneous determination of the studied AIIRAs–amlodipine mixtures in pharmaceutical dosage form

Drug	Dosage form	% Recovery^(a)^ ± SD		t-value ^(b)^	F-value ^(b)^	Refs.
		Proposed method	Reported method			
OLM–AML	Erastapex ^®^					
OLM		97.31 ± 1.02	99.15 ± 2.24	1.84	4.82	[53]
AML		101.21 ± 1.27	102.11 ± 0.85	1.44	2.24	[15]
TLM–AML	Chartoreg ^®^					
TLM		97.74 ± 1.94	98.65 ± 1.95	0.81	1.02	[15]
AML		102.42 ± 1.10	102.11 ± 0.85	1.66	0.55	[15]
CAN–AML						
CAN	Candalkan^®^	99.39 ± 1.62	98.26 ± 2.44	0.94	2.28	[14]
AML	Norvasc^®^	101.76 ± 1.21	102.11 ± 0.85	0.58	2.03	[15]
LOS–AML						
LOS	Amosar^®^	101.25 ± 1.12	100.14 ± 2.15	1.11	3.65	[14]
AML	Norvasc^®^	101.77 ± 1.39	102.11 ± 0.85	0.51	2.68	[15]
IRB–AML						
IRB	Aprovel^®^	98.52 ± 1.67	99.17 ± 1.77	0.76	2.31	[14]
AML	Norvasc^®^	100.88 ± 1.49	102.11 ± 0.85	1.76	3.08	[15]

(a) Average of six determinations ± standard deviation

(b) Theoretical values at 95% confidence limit; are t = 2.262, F = 5.05

### Application of the proposed HPTLC method in spiked human plasma

**Fig. 7:**
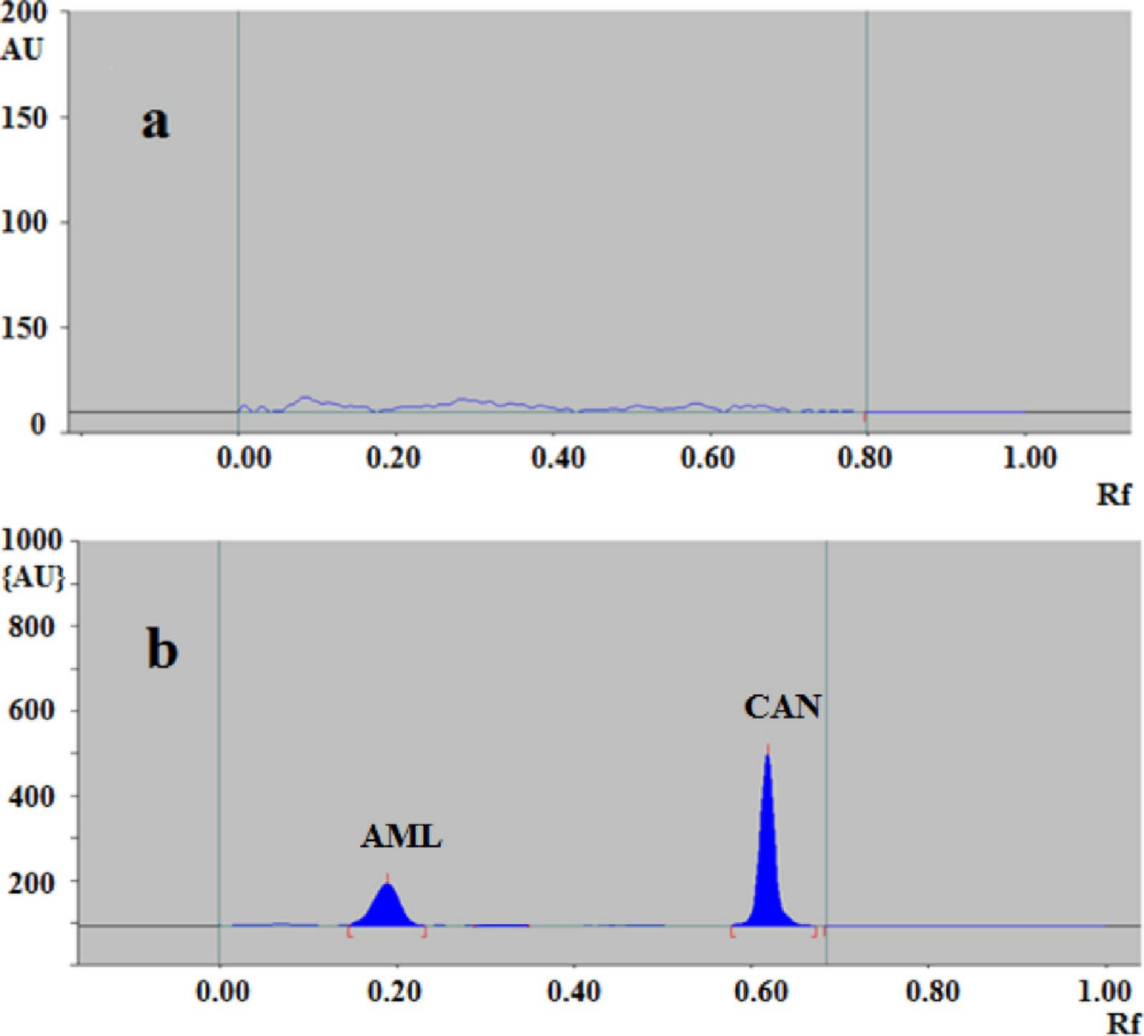
HPTLC densitogram of plasma sample (**a**) without spiking with AML and CAN (**b**) plasma sample spiked with (600 ng/band) of AML and (900 ng/band) of CAN

The proposed method was successfully used for the determination of the studied drugs in spiked human plasma. Six replicate measurements were made of three distinct concentrations of each drug utilizing the chromatographic conditions mentioned before. Since it was difficult to get blood from hypertensive patients who had received the examined medicines, we decided to test our suggested method on samples of spiked human plasma to see if it could detect the studied drugs’ residual levels in human plasma after delivery. Blank plasma sample without spiking with the studied drugs was analyzed as shown in (Fig. 7a). Then, the plasma samples were spiked with the studied drug mixture at 3 different concentration levels for each drug as illustrated in (Fig. 7b). The high estimated mean percent recovery indicates that the existing methodology for examining these antihypertensive medications in spiked plasma is effective and reliable (Table 6). The recovery percentages ranged from 93.31 to 107.92 and standard deviations were in the range of (0.82–2.54).

**Table 6:** Application of the proposed HPTLC method with UV detection for simultaneous determination of the studied AIIRAs–Amlodipine mixtures in spiked human plasma

Studied mixtures	AIIRAs OLM, TLM, CAN, LOS, IRB			AML		
	Conc. (ng/band)	Amount found (ng/band)	% Recovery (a) ± SD	Conc. (ng/band)	Amount found (ng/band)	% Recovery (a) ± SD
OLM–AML	150	144.19	96.13 ± 2.13	120	126.69	105.57 ± 1.20
	300	316.20	105.40 ± 1.70	240	248.01	103.34 ± 2.61
	420	411.49	97.97 ± 1.43	360	369.59	102.66 ± 1.81
TLM–AML	150	142.28	94.85 ± 1.18	120	127.17	105.98 ± 2.40
	420	404.13	96.22 ± 1.92	360	349.35	97.04 ± 1.16
	600	579.98	96.66 ± 2.54	480	458.44	95.51 ± 0.89
CAN–AML	150	157.64	105.09 ± 0.82	120	112.97	94.14 ± 1.44
	300	282.11	94.04 ± 1.79	360	339.60	94.33 ± 2.37
	420	437.67	104.21 ± 1.50	600	563.38	93.90 ± 1.98
LOS–AML	120	126.24	105.20 ± 1.73	90	83.98	93.31 ± 2.16
	270	282.08	104.47 ± 1.12	270	256.85	95.13 ± 0.91
	420	447.10	106.45 ± 2.53	420	404.65	96.35 ± 2.37
IRB–AML	120	114.48	95.40 ± 1.06	90	97.13	107.92 ± 1.45
	210	219.36	104.46 ± 1.38	210	216.92	103.29 ± 1.96
	360	345.85	96.07 ± 2.11	360	378.32	105.09 ± 2.53

(a) Average of six determinations at each concentration level

We compared the obtained recovery percentages in human plasma with the reported HPLC methods ranged from 97.51 to 99.12 for AML and 97.88 to 101.07 for CAN [44, 45]. Also, these methods require long term and exhausting sample preparation and several tests of stability, this proposed HPTLC method is simple, cost-effective and not require sample preparation.

### Comparison of analytical methods for studied anti-hypertensive drugs

Various analytical techniques have been developed for the quantification of anti-hypertensive drugs, each with distinct advantages and limitations. Methods such as HPLC, HPTLC, spectrophotometry, and electrochemical analysis have been utilized in pharmaceutical and biological matrices. A comparative summary of these methods is presented in Table S2, providing insights into their advantages and disadvantages.

### Evaluation the greenness of the proposed HPTLC method

Eco-scale can be assessed [46–50]. An Eco-scale penalty paradigm is used to evaluate the ecology of the method. The Eco-scale study’s result is a 100-point deviation penalty (“ideal green analysis”). The dots represent the hazards that were used in the analysis. The more valuable the technique, the more environmentally friendly it is. The recommended method substitutes water for organic solvents. Furthermore, energy-intensive processes like heating that consume more than 0.1 kWh per sample were avoided. With an eco-scale score of 79, the recommended methods were classified as good green practices (Table 7).

**Table 7:** Evaluation the greenness of the proposed HPTLC method with UV detection using the eco-scale score approach

Item	Parameter	Word sign	PP sign
Technique	TLC	LSH	1
Reagent(s)	Methanol		3
	Ethyl acetate		2
	Toluene		3
	Acetone		2
	Acetic acid		4
Solvent	Methanol		3
Energy consumption (kW/h)	≤ 0.1		0
Occupational hazards			0
Waste	1–10 mL		3
Heating	No heating		0
Temperature	Room temperature		0
Cooling	No cooling		0
Total penalty points (TPPs)			21
Eco-scale total score	= 100−TPP		79

LSH for the Less severe hazard, and TPPs for the Total penalty points

Another new tool for assessing greenness is the Analytical Greenness Calculator (AGREE), which offers a straightforward, adaptable, and comprehensive approach [51]. The calculator’s software is readily available, and it’s easy to interpret its results. The output, represented by a pictogram, displays the final score in the center. A score of roughly one (0.68) and the color green at the pictogram center suggest a high level of greenness in the procedure.

Furthermore, each of the 12 Green Analytical Chemistry (GAC) ideas is integrated into the pictogram in the shape of segments of color. Red is the least green color, and thankfully, there are only one shade of red. Dark green, on the other hand, is the greenest color.

Fig. S5 displays the outcomes of the AGREE for the suggested HPTLC method.

The GAPI [52] is a new tool that recently became available. It is thought to be among the most innovative and finest methods for assessing environmental friendliness. This tool successfully fixes the issues with the previously stated utilities. The GAPI tool uses pictograms to categorize the level of greenness at each stage of the analysis process. Yellow, red, and green stand for low, medium, and high environmental impacts, respectively.

The suggested HPTLC under investigation complies with most of GAPI’s standards. since they stand for untreated waste and offline sampling, respectively. The GAPI pentagrams showed that the suggested approach achieves a satisfactory green level, as seen by the 9 green, 3 yellow, and 3 red shaded areas in Fig. S5.

## Conclusion

The proposed high-performance thin-layer chromatographic (HPTLC) method with UV detection provides a quick, user-friendly, cost-effective, and sensitive approach for the simultaneous determination of Amlodipine (AML) and the studied Angiotensin II Receptor Antagonists (AIIRAs). This method allows for the detection of both drugs in a single run at one scanning wavelength. Notably, there are no published HPTLC methods that simultaneously determine AML with various AIIRA members. Due to the high sensitivity, simplicity, and selectivity of the HPTLC method, this approach successfully analyzed AML and the studied AIIRAs in human plasma without interference from plasma constituents. This methodology can be applied in future pharmacokinetic studies and can effectively determine the plasma concentrations of the studied drugs, owing to the excellent recovery of the drugs from plasma.

## Supplementary Information

Below is the link to the electronic supplementary material.

Supplementary Material 1.

## Data Availability

All data generated or analyzed during this study are included in this published article.
