# Supplementary material for: High-performance thin-layer chromatographic method for simultaneous determination of some Angiotensin II Receptor Antagonists with amlodipine in spiked human plasma with UV detection
Source: BMC Chem. 2026 Mar 23;20(1):79. doi: 10.1186/s13065-026-01765-6 (PMC13064381; doi:10.1186/s13065-026-01765-6)
Supplement: Supplementary file 1 — Supplementary Material 1. [file 13065_2026_1765_MOESM1_ESM.docx]

**Table S1: Different mobile phases composition trials for the separation of some AIIRAs – amlodipine mixtures and the corresponding Rf:**

| **Mobile phase** | **OLM** | **TLM** | **CAN** | **LOS** | **IRB** | **AML** |
| --- | --- | --- | --- | --- | --- | --- |
| Dichloromethane: methanol: acetic acid  4: 5.5: 0.5 | At the solvent front | | | | | 0.72 |
| Dichloromethane: methanol: acetic acid  7: 2.7: 0.3 | At the solvent front | | | | | 0.71 |
| Toluene: ethyl acetate: methanol: acetic acid  4: 4: 1.8: 0.2 | 0.65 | 0.68 | 0.73 | 0.65 | 0.67 | 0.09 |
| Toluene: ethyl acetate: methanol: acetic acid  3.5: 4: 2.3: 0.2 | 0.78 | 0.77 | 0.83 | 0.76 | 0.80 | 0.14 |
| Toluene: ethyl acetate: acetone: acetic acid  3.5: 4: 2.3: 0.2 | 0.58 | 0.46 | 0.72 | 0.60 | 0.64 | At baseline |
| Toluene: ethyl acetate: acetone: acetic acid  3: 3: 3.8: 0.2 | 0.69 | 0.67 | 0.76 | 0.69 | 0.72 | At baseline |
| Toluene: ethyl acetate: methanol: acetone: acetic acid 3: 4: 2.3: 0.5: 0.2 | 0.81 | 0.81 | 0.84 | 0.76 | 0.81 | 0.15 |
| Toluene: ethyl acetate: methanol: acetone: acetic acid 3: 4: 2: 0.5: 0.5 | 0.83 | 0.82 | 0.88 | 0.83 | 0.83 | 0.19 |
| Toluene: ethyl acetate: methanol: acetone: acetic acid 4: 3: 1: 0.5: 1.5 | 0.75 | 0.68 | 0.85 | 0.79 | 0.81 | 0.30 |
| Toluene: methanol: acetic acid  6: 2: 2 | No resolution between the drugs | | | | | |
| Toluene: ethyl acetate: methanol: acetone: acetic acid 5: 1.5: 1: 0.5: 2 | 0.72 | 0.75 | 0.84 | 0.76 | 0.81 | 0.5 |
| Toluene: ethyl acetate: methanol: acetonitrile: acetic acid 6: 1.5: 1: 0.5: 1 | 0.52 | 0.55 | 0.63 | 0.54 | 0.60 | 0.26 with tailing |
| Toluene: ethyl acetate: methanol: acetonitrile: acetic acid 6: 1.5: 1: 1: 0.5 | 0.57 | 0.58 | 0.63 | 0.59 | 0.61 | 0.21 with tailing |
| Toluene: ethyl acetate: methanol: acetonitrile: acetic acid 4.5: 1.5: 1: 1: 2 | 0.74 | 0.78 | 0.82 | 0.80 | 0.80 | 0.46 |
| Toluene: ethyl acetate: acetonitrile: acetic acid 4.5: 2.5: 1: 2 | 0.55 | 0.68 | 0.80 | 0.70 | 0.65 | 0.17 with tailing |
| Toluene: ethyl acetate: methanol: acetone: acetic acid 6: 1.5: 1: 0.5: 1 | **0.50** | **0.40** | **0.62** | **0.69** | **0.73** | **0.22** |

**Table S2: A table summarizing previous analytical methods for the studied anti-hypertensive drugs with their advantages/disadvantages:**

| **Method** | **Drugs** | **Advantages** | **Disadvantages** | **Reference** |
| --- | --- | --- | --- | --- |
| UV spectrophotometry | OLM, TLM and AML | * Rapid and simple  * Non- Destructive  * Low cost  * Saving time  * Good reproducibility | * Limited to UV absorbing compounds  * Restricted to pharmaceutical formulation application not human plasma  * Possible instrumental error | [[1](#_ENREF_1), [2](#_ENREF_2)] |
| spectrofluorometry | TLM, CAN, LOS, IRB and AML | * High sensitivity  * High selectivity  * Rapid analysis  * Enhanced signal-to- noise ratio  * Application versatility | * Limited to fluorescent compounds  * Photobleaching  * Quenching effect  * Instrumental cost | [[3-5](#_ENREF_3)] |
| TLC | TLM, CAN, LOS and AML | * Cost-effective  * Easy to perform  * Simultaneous analysis  * Simple and quick  * Application versatility  * Visual detection  * Flexible mobile phases | * Non- automated process  * Short plate lifespan  * Slow development for some samples | [[6-8](#_ENREF_6)] |
| HPLC | OLM, TLM and IRB | * Reproudicble and reliable  * Compatible with multiple detectors  * Quantitative and qualitative analysis  * High sensitivity and selectivity | * High cost  * Complex operation  * Column degradation  * Solvent consumption and waste  * Limited to liquid soluble compounds  * Time consuming | [[9](#_ENREF_9), [10](#_ENREF_10)] |
| Voltammetry | AML | * High sensitivity and selectivity  * Small sample volume required  * Applicable to wide range of compounds | * Electrode fouling and contamination  * Less widely used than HPLC or spectroscopy  * Interference from other electroactive species | [[11-13](#_ENREF_11)] |


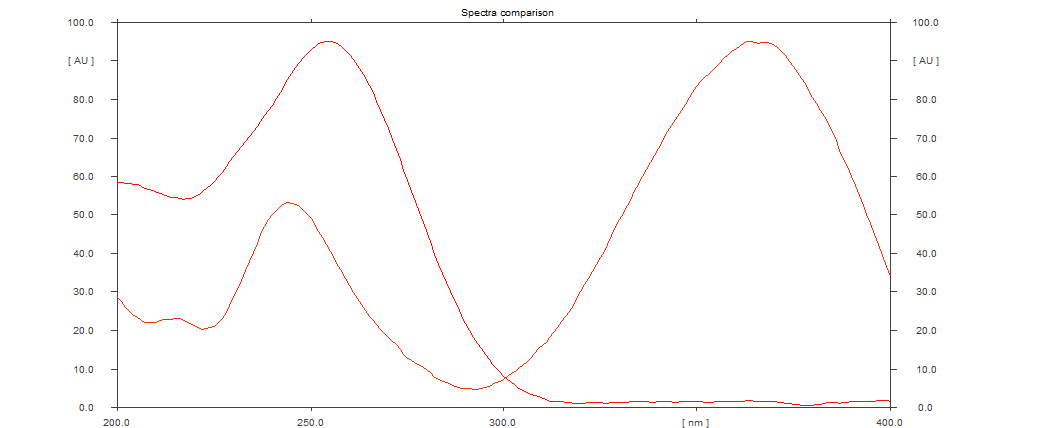


**Fig S1.**  Absorption spectra of Amlodipine and Olmesartan


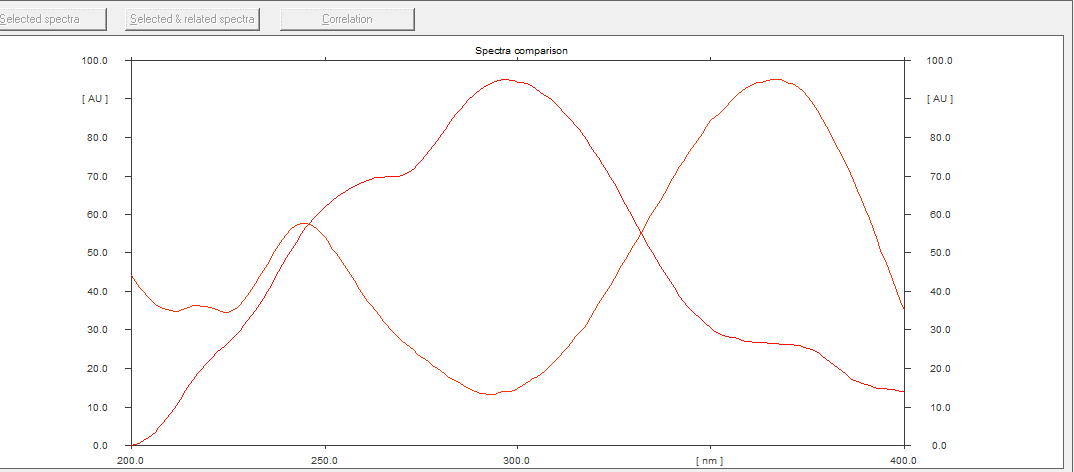


**Fig S2.**  Absorption spectra of Amlodipine and Telmisartan


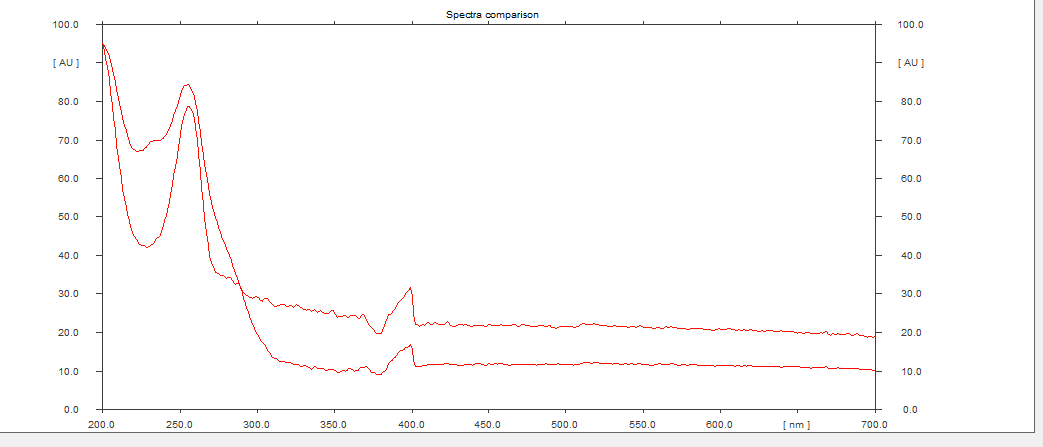


**Fig S3.**  Absorption spectra of Amlodipine and Losartan


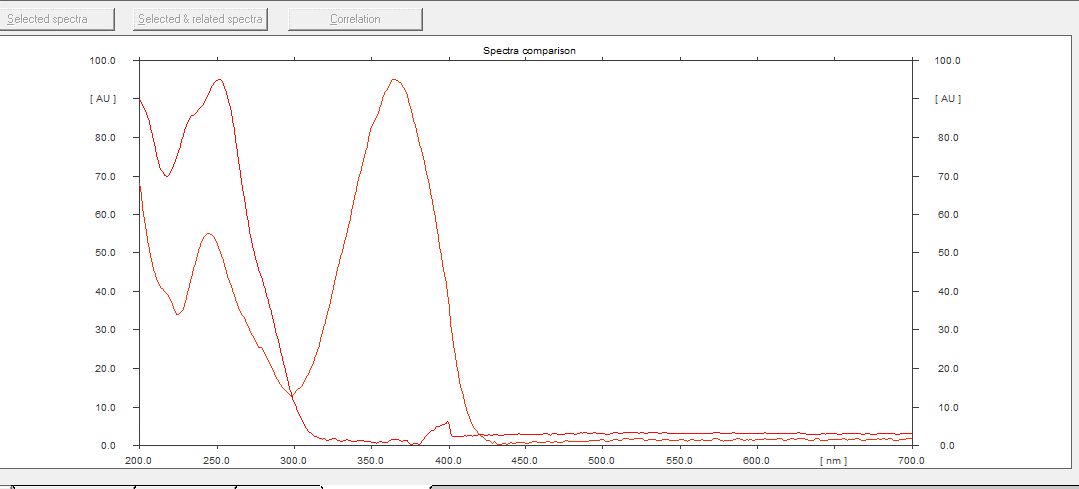


**Fig S4.**  Absorption spectra of Amlodipine and Irbesartan


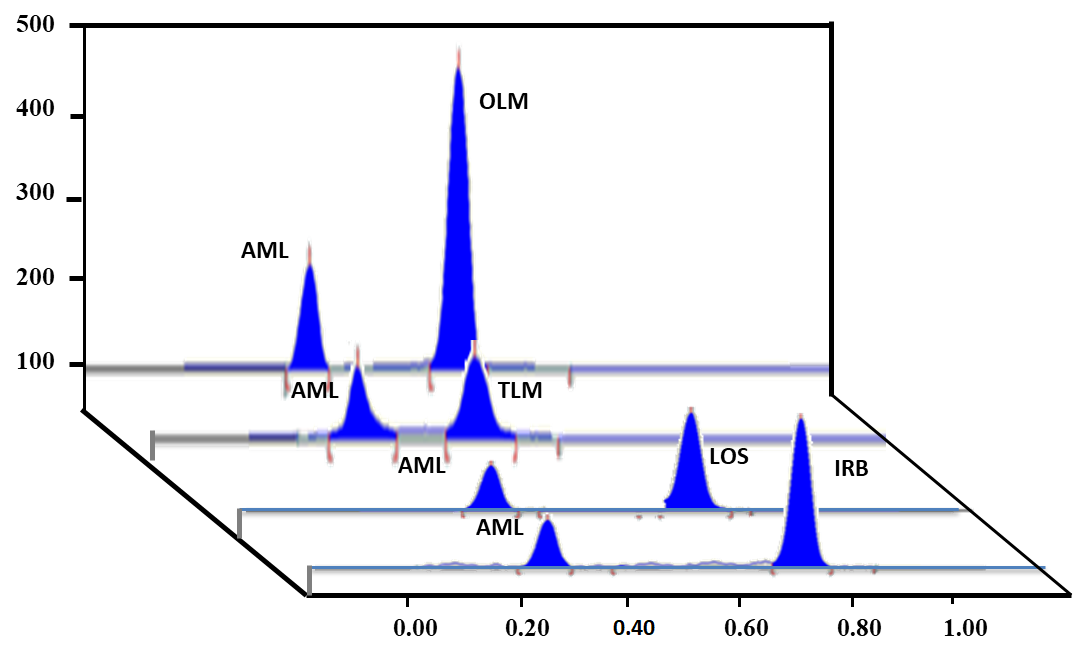


**Fig S5.**  HPTLC densitogram of mixture containing

600 ng/band of AML and 900 ng/band of OLM.

480 ng/band of AML and 600 ng/band of TLM.

270 ng/band of AML and 270 ng/band of LOS.

420 ng/band of AML and 420 ng/band of IRB.


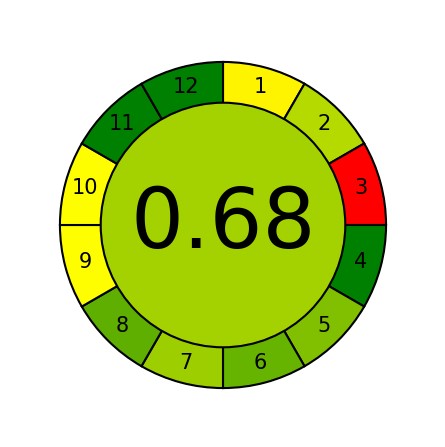

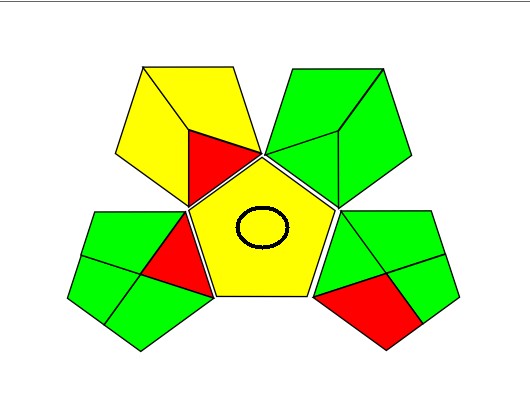


**(A) (B)**

**Fig S6.** Evaluation of the greenness of the proposed HPTLC method using AGREE (A) and GAPI (B) methods


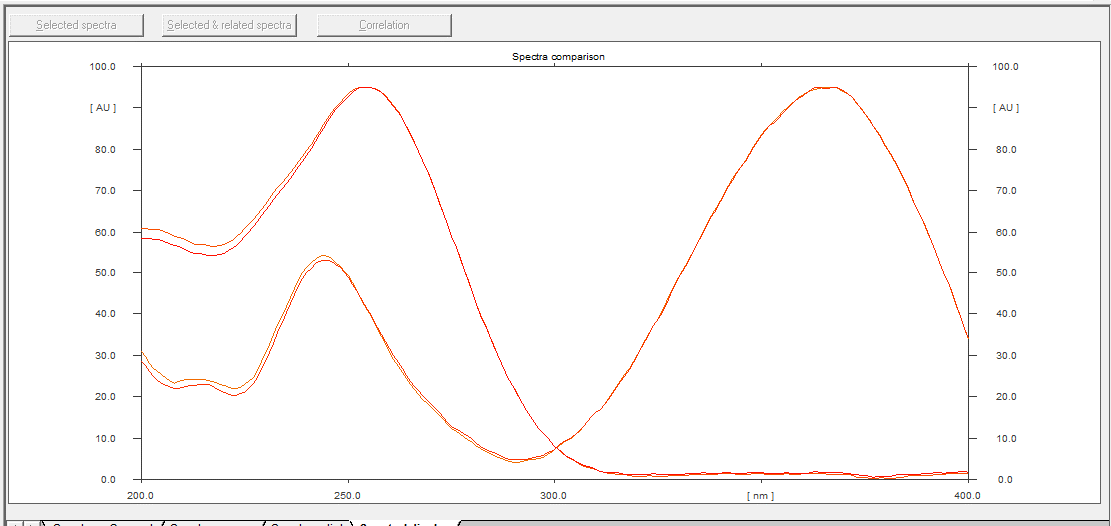


Figure S7. Absorption spectra of standard solution and pharmaceutical

extract of Erastapex® tablets (2) containing OLM (400 ng/band) and

AML (100 ng/band).


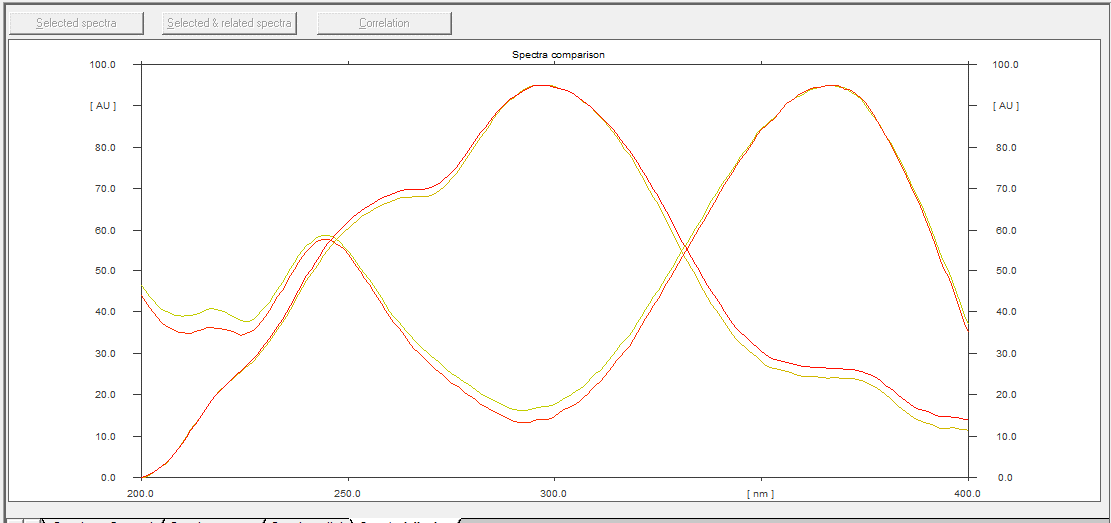


Figure S8. Absorption spectra of standard solution and pharmaceutical

extract of Chartoreg CO®® tablets (2) containing TELM (400 ng/band) and

AML (100 ng/band).

1. Patil PS, Kulkarni PD, Burkul MS, More HN, Pishawikar SA: **Simultaneous estimation of amlodipine besylate and olmesartan medoxomil by first order derivative spectroscopy from tablet**. *International Journal of PharmTech Research* 2011, **3**(2):668-675.

2. Pandey A, Sawarkar H, Singh M, Kashyap P, Ghosh P: **UV-spectrophotometric method for estimation of telmisartan in bulk and tablet dosage form**. *International Journal of ChemTech Research* 2011, **3**(2):657-660.

3. Abdelmonem AA, Ragab GH, Hashem HA, Bahgat EA: **Spectrofluorimetric and spectrophotometric determination of irbesartan and bisoprolol hemifumarate independently in their tablets**. *Pharmaceutical and Biosciences Journal* 2016:43-52.

4. El-Shaboury SR, Hussein SA, Mohamed NA, El-Sutohy MM: **Spectrofluorimetric method for determination of some angiotensin II receptor antagonists**. *Journal of pharmaceutical analysis* 2012, **2**(1):12-18.

5. Belal TS, Mahrous MS, Abdel‐Khalek MM, Daabees HG, Khamis MM: **Validated spectrofluorimetric determination of two pharmaceutical antihypertensive mixtures containing amlodipine besylate together with either candesartan cilexetil or telmisartan**. *Luminescence* 2014, **29**(7):893-900.

6. Saeed S, Nadim AH, Yehia AM, Moustafa AA: **A versatile high-performance thin-layer chromatographic method for the simultaneous determination of five antihypertensive drugs: method validation and application to different pharmaceutical formulations**. *JPC–Journal of Planar Chromatography–Modern TLC* 2021, **34**:467-477.

7. Deshmukh TB, Deo SS, Inam FS, Lambat TL: **Development and validation of novel HPTLC method for the simultaneous estimation of amlodipine besylate and telmisartan in tablet dosage form using ICH Q2 (R1) directions**. *Journal of the Chinese Advanced Materials Society* 2018, **6**(4):329-340.

8. HINGE MA, PATEL D: **Simultaneous Estimation of Amlodipine Besylate and Candesartan Cilexitil by HPTLC Method in their Combined Dosage Form**. *International Journal of Pharmaceutical Research (09752366)* 2021, **13**(2).

9. Alhazmi HA, Alnami AM, Arishi MA, Alameer RK, Al Bratty M, Rehman ZU, Javed SA, Arbab IA: **A fast and validated reversed-phase HPLC method for simultaneous determination of simvastatin, atorvastatin, telmisartan and irbesartan in bulk drugs and tablet formulations**. *Scientia Pharmaceutica* 2018, **86**(1):1.

10. Hamrapurkar PD, Gadapayale KK: **Optimization and validation of RP-HPLC stability indicating method for determination of olmesartan medoxomil and its degraded product**. *International Journal of Applied Science and Engineering* 2013, **11**(2):137-147.

11. Khorshed AA, Khairy M, Banks CE: **Electrochemical determination of antihypertensive drugs by employing costless and portable unmodified screen-printed electrodes**. *Talanta* 2019, **198**:447-456.

12. Khairy M, Khorshed AA, Rashwan FA, Salah GA, Abdel-Wadood HM, Banks CE: **Simultaneous voltammetric determination of antihypertensive drugs nifedipine and atenolol utilizing MgO nanoplatelet modified screen-printed electrodes in pharmaceuticals and human fluids**. *Sensors and Actuators B: Chemical* 2017, **252**:1045-1054.

13. Khairy M, Khorshed AA: **Simultaneous voltammetric determination of two binary mixtures containing propranolol in pharmaceutical tablets and urine samples**. *Microchemical Journal* 2020, **159**:105484.
